# Elizabethkingia meningoseptica With Targeted Environmental Surveillance in a Tertiary Care Hospital: A Retrospective Cohort Study

**DOI:** 10.7759/cureus.87095

**Published:** 2025-07-01

**Authors:** Garima Mittal, Rajender Singh, Manish Mittal, Mani Pant, Barnali Kakati, Saikat Patra

**Affiliations:** 1 Microbiology, Himalayan Institute of Medical Sciences, Swami Rama Himalayan University, Dehradun, IND; 2 Neurology, Himalayan Institute of Medical Sciences, Swami Rama Himalayan University, Dehradun, IND; 3 Neonatology, Himalayan Institute of Medical Sciences, Swami Rama Himalayan University, Dehradun, IND

**Keywords:** drug resistance, elizabethkingia meningoseptica, infection control, sepsis, ventilator-associated pneumonia

## Abstract

Introduction

*Elizabethkingia meningoseptica* (*E. meningoseptica*​​​) ​​​is a multidrug-resistant, non-fermenting Gram-negative bacillus increasingly associated with nosocomial infections, particularly in immunocompromised and critically ill patients. Its intrinsic resistance to multiple antibiotics limits treatment options and contributes to adverse clinical outcomes.

Aim and objective

The study aimed to investigate the clinical and microbiological characteristics of *E. meningoseptica* infections among ICU/NICU patients and to explore potential environmental sources through hospital-based surveillance. The objectives of this study were to describe the clinical and demographic profiles of ICU/NICU patients with *E. meningoseptica* infection; to assess the associated risk factors contributing to these infections; to analyze the antimicrobial susceptibility patterns of the isolated strains using standardized microbiological methods; to evaluate treatment outcomes including morbidity, mortality, and duration of ICU/NICU stay; and to identify potential environmental reservoirs of *E. meningoseptica* through surveillance activities coordinated by the Hospital Infection Control Committee (HICC).

Methods

A retrospective cohort study was conducted over 18 months (July 2023 to October 2024) at a tertiary care hospital. A total of 15 patients with culture-confirmed *E. meningoseptica* infection were included. Demographic, clinical, microbiological, and treatment data were collected. Identification and antimicrobial susceptibility testing were performed using the Vitek-2 automated identification system (bioMérieux, Marcy-l'Étoile, France). HICC conducted routine and targeted environmental sampling as per policy protocol.

Results

Among the 15 patients with *E. meningoseptica* infection, nine (60.0%) were adults, and 11 (73.3%) were male individuals. Sepsis was reported in nine (60.0%), and both ventilator-associated pneumonia and septic shock occurred in six patients each (40.0%). Hypertension and diabetes mellitus were present in six (40.0%) and three (20.0%) patients, respectively. Two neonates (13.3%) with low birth weight and prematurity required NICU admission. Outcomes included mortality in six patients (40.0%), discharge in five (33.3%), and four (26.7%) leaving against medical advice. Endotracheal secretions were the most frequent specimen (46.7%), followed by tracheostomy secretions (33.3%) and blood cultures (13.3%).

All isolates (100%) were resistant to carbapenems, third-generation cephalosporins, aztreonam, and piperacillin-tazobactam. Resistance to gentamicin, colistin, and cotrimoxazole was observed in 73.3%, 66.7%, and 46.7% of isolates, respectively. Highest susceptibility was seen with minocycline (66.7%), vancomycin (60.0%), and fluoroquinolones (53.3%).

Environmental surveillance, conducted from January to March 2023 as part of routine as well as targeted HICC monitoring (as an outbreak of suspected pathogens was suspected from the NICU and ICU), included 98 samples from high-risk ICU/NICU sites such as sinks, ventilators, humidifiers, and suction units. *E. meningoseptica* was identified in one sample (1.02%), isolated from a NICU sink.

Conclusion

*E. meningoseptica* infections are associated with high resistance and mortality. Minocycline, vancomycin, and fluoroquinolones may be effective. Early detection, targeted therapy, and HICC-led surveillance are essential for control.

## Introduction

Elizabethkingia is a ubiquitous environmental organism [1]. The most frequently isolated species is *Elizabethkingia meningoseptica *(*E. meningoseptica*), and it was first identified in 1959 by the American bacteriologist Elizabeth O. King and was later recognized as a causative agent of neonatal septicemia [2]. This Gram-negative, non-motile, non-fermenting rod is both oxidase- and catalase-positive. It is associated with a variety of infections in both neonatal and adult populations. In neonates, *E. meningoseptica* is a known cause of meningitis, pneumonia, bacteremia, and sepsis [3]. Meningitis due to this pathogen has a reported mortality rate of approximately 57%, with long-term complications such as hydrocephalus, deafness, and developmental delay [3]. In adults, the infection predominantly occurs in nosocomial settings, affecting both immunocompetent and immunocompromised individuals. Common clinical manifestations include pneumonia, endocarditis, postoperative bacteremia, abdominal infections, bronchitis, and meningitis [3,4]. Several risk factors contribute to* E. meningoseptica* infections, including prolonged immunosuppression, underlying comorbidities, extended hospital stays, prior use of broad-spectrum antibiotics (such as third-generation cephalosporins and carbapenems), and the presence of invasive medical devices like central venous catheters [4].

*E. meningoseptica* is widely present in hospital environments and has been implicated in severe nosocomial infections, often linked to contaminated medical equipment. The organism has been isolated from various sources, including hospital water systems, sinks, taps, saline solutions used for flushing, disinfectants, and a range of medical devices such as feeding tubes, arterial catheters, and respirators. Environmental studies have demonstrated its ability to survive in chlorinated municipal water, allowing it to colonize sink basins, taps, intubation tubes, humidifiers, neonatal incubators, ice chests, and syringes. These contaminated surfaces and devices can serve as persistent reservoirs, contributing to hospital-acquired infections [5-7].

Elizabethkingia exhibits a distinct antimicrobial susceptibility profile, showing sensitivity to only a limited number of commonly used antibiotics, including piperacillin-tazobactam and minocycline [8,9]. However, it demonstrates resistance to many routinely used antimicrobials, such as aminoglycosides, beta-lactams, tetracyclines, and carbapenems. The primary mechanism of resistance to beta-lactam antibiotics is attributed to the production of extended-spectrum-beta-lactamases (ESBLs) and carbapenem-hydrolysing metallo-beta-lactamases. This study aims to retrospectively analyze the clinical presentation, risk factors, antimicrobial susceptibility patterns, and outcomes of *E. meningoseptica *infections, and to identify potential environmental reservoirs through Hospital Infection Control Committee (HICC)-led surveillance.

## Materials and methods

Study design

This retrospective cohort study was conducted at a tertiary care hospital over 18 months, from July 2023 to October 2024. The study focused on patients admitted to the intensive care unit (ICU) and neonatal intensive care unit (NICU) with microbiologically confirmed *E. meningoseptica* infections.

Inclusion criteria

Patients were included if they had a culture-confirmed infection with *E. meningoseptica* and were admitted to either the ICU or NICU during the study period. Only those with complete clinical and microbiological records were eligible for analysis.

Exclusion criteria

Patients were excluded if medical records were incomplete or if the infection was polymicrobial and *E. meningoseptica* was not the dominant pathogen.

Ethical approval

The study protocol was reviewed and approved by the Research Committee of the Himalayan Institute of Medical Sciences, Swami Rama Himalayan University, Dehradun, under approval number SRHU/HIMS/RC/2025/167, dated June 6, 2025. All procedures were conducted per institutional and national ethical guidelines, and patient confidentiality was strictly maintained throughout the study.

Microbiological diagnosis

Clinical specimens collected from suspected cases were processed using standard microbiological techniques. Identification and antimicrobial susceptibility testing of *E. meningoseptica* isolates were carried out using the Vitek-2 automated identification system (bioMérieux, Marcy-l'Étoile, France). Additional susceptibility to colistin and vancomycin was assessed. Antimicrobial susceptibility was interpreted according to the Clinical and Laboratory Standards Institute (CLSI) guidelines applicable at the time of testing [10].

Environmental surveillance

To identify potential environmental sources, the HICC conducted routine as well as targeted surveillance in view of suspecting a pathogen outbreak in the ICU and NICU. A total of 98 samples were collected, comprising 63 surface swabs and 35 water samples from high-risk areas such as ICU and NICU sinks, taps, humidifier vents, bed surfaces, breast pumps, ventilator surfaces, cardiac table surfaces, and water outlets. All samples were pre-enriched in trypticase soy broth and incubated at 35-37°C for 48 hours. Subsequent subcultures were performed on blood agar and MacConkey agar, and incubated for 18-24 hours. Colonies exhibiting moist, grey, slightly hemolytic features were subjected to species confirmation and antimicrobial susceptibility testing using the Vitek-2 system.

Data analysis

Data on demographics, clinical features, antimicrobial regimens, outcomes, and microbiological profiles were extracted and compiled into a standardized dataset. Descriptive statistics were used to summarize findings, reported as frequencies and percentages (n (%)). Given the small sample size, no inferential statistical tests were applied. Subgroup patterns (e.g., ICU vs. NICU) and antimicrobial resistance trends were explored descriptively to support clinical interpretation. Statistical analyses were performed using IBM SPSS Statistics for Windows, Version 25 (IBM Inc., Armonk, NY, USA).

## Results

Patient demographics

Between July 2023 and October 2024, 15 patients were diagnosed with *E. meningoseptica* infection at our tertiary care hospital. All were admitted to critical care settings, including 13 in the ICU and two in the NICU. The age of affected individuals ranged from 23 days to 81 years, with adults accounting for nine cases (60.0%). A male predominance was noted, with 11 patients (73.3%) being of the male gender.

Clinical characteristics and risk factors

Sepsis was the most common provisional diagnosis, reported in nine patients (60.0%), followed by ventilator-associated pneumonia and septic shock, each in six patients (40.0%). Comorbidities included hypertension in six patients (40.0%) and diabetes mellitus in three (20.0%). Two neonates (13.3%) presented with low birth weight and prematurity.

Clinical outcomes

Among the 15 cases, mortality occurred in six patients (40.0%). Five patients (33.3%) were discharged, and four (26.7%) left against medical advice (LAMA). Due to the small sample size, no inferential statistical analysis was performed; however, most non-survivors had underlying comorbidities such as hypertension and diabetes.

Specimen distribution

The most frequently collected specimens were endotracheal tube secretions (seven cases; 46.7%), followed by tracheostomy secretions (five cases; 33.3%) and blood cultures (two cases; 13.3%). One isolate was recovered from a urine sample.

Antimicrobial therapy

Empirical therapy predominantly included meropenem (14 patients; 93.3%) and amikacin (11 patients; 73.3%). Definitive treatment varied based on antimicrobial susceptibility and clinical response, with vancomycin administered in six patients (40.0%), aminoglycosides in four (26.7%), and cefoperazone-sulbactam in two (13.3%). Additional agents such as levofloxacin, minocycline, linezolid, and ceftazidime-avibactam were used selectively in specific cases (Table 1).

**Table 1:** Clinical characteristics and microbiological profile of patients with Elizabethkingia meningoseptica infections LOS: length of stay; M: male; F: female; RDS: respiratory distress syndrome; VAP: ventilator-associated pneumonia; LBW: low birth weight; LAMA: left against medical advice; ET: endotracheal tube; TT: tracheostomy tube; CA: carcinoma; DM-2: diabetes mellitus type 2; HTN: hypertension; SAH: subarachnoid hemorrhage; ICA: internal carotid artery; RTA: road traffic accident; AF: atrial fibrillation; AKI: acute kidney injury; NICU: neonatal intensive care unit; VP shunt: ventriculo-peritoneal shunt; LVEF: left ventricular ejection fraction

Case no.	LOS (days)	Age	Gender	Provisional diagnosis	Risk factors	Outcome	Specimen	Empirical treatment	Definitive treatment
1	30	23 days	M	RDS/pneumonia-VAP/sepsis	LBW, preterm, anemia	Discharge	ET secretion	Meropenem, amikacin	Vancomycin
2	30	11 years	M	Status epilepticus? Sepsis	Vitamin D deficiency, seizures	LAMA	TT secretion	Colistin, meropenem, vancomycin	Colistin
3	27	55 years	M	Septic shock, VAP	CA-lung, DM-2, HTN	Death	ET secretion	Meropenem	Vancomycin
4	13	62 years	M	Diffuse SAH with left ICA bifurcation	HTN	Death	ET secretion	Meropenem, amikacin	Vancomycin
5	27	47 years	M	VAP	RTA - head injury	LAMA	TT secretion	Colistin, meropenem, vancomycin	Cefoperazone-sulbactam
6	30	81 years	F	Shock, sepsis - VAP	HTN	Death	Blood	Meropenem, amikacin	Cefoperazone-sulbactam gentamicin
7	24	58 years	F	Shock, sepsis - VAP	DM-2, HTN	Death	TT secretion	Colistin, meropenem, vancomycin	Levofloxacin
8	40	74 years	F	Shock, sepsis - VAP	Dilated cardiomyopathy, AF	Death	ET secretion	Meropenem, amikacin	Vancomycin
9	95	77 years	M	Status epilepticus	Dilated cardiomyopathy, AF	Discharge	ET secretion	Meropenem, amikacin	Vancomycin, Augmentin
10	12	62 years	M	Uremic sepsis, AKI	DM-2, multiple myeloma	LAMA	ET secretion	Meropenem, amikacin	Ceftazidime + avibactam, aztreonam
11	44	57 years	M	Shock, sepsis/excision of giant vestibular schwannoma	Ventriculo-peritoneal shunt	Death	TT secretion	Meropenem, amikacin	Vancomycin
12	57	9m 6 days	M	Shock, sepsis, meningitis	LBW, preterm, RDS	Admitted in NICU	Blood	Amikacin, ceftriaxone	Amikacin, ceftriaxone, Ciprofloxacin
13	20	37 years	M	Right putamen bleed	Surgery, HTN	Discharge	TT secretion	Meropenem, amikacin	Ciprofloxacin, amikacin
14	10	40 years	F	Sepsis, VAP	HTN	LAMA	ET secretion	Meropenem, amikacin	Minocycline, tobramycin
15	6	79 years	M	Sick sinus syndrome	LVEF-56%	Discharge	Urine	Meropenem, amikacin	Linezolid, cefuroxime

Among 15 *E. meningoseptica* isolates, 100% (15/15) were resistant to carbapenems (imipenem and meropenem) as well as to cephalosporins (ceftazidime and cefepime), aztreonam, and piperacillin-tazobactam, confirming the multidrug-resistant nature of the organism. Resistance was also high for gentamicin in 11 (73.3%) isolates, colistin in 10 (66.7%), and cotrimoxazole in seven (46.7%). Among the agents showing better efficacy, minocycline demonstrated susceptibility in 10 (66.7%) isolates, vancomycin in nine (60%), and both levofloxacin and Ciprofloxacin in eight (53.3%) isolates each. These findings underscore the limited therapeutic choices and emphasize the importance of susceptibility-guided treatment (Table 2).

**Table 2:** Antimicrobial susceptibility profile of Elizabethkingia meningoseptica isolates (n-15) PIT: piperacillin-tazobactam; CAZ: ceftazidime; CPS: cefoperazone-sulbactam; CPM: cefepime; AT: aztreonam; IPM: imipenem; MER: meropenem; AK: amikacin; GEN: gentamicin; CIP: ciprofloxacin; LE: levofloxacin; Col: colistin; COT: cotrimoxazole; VA: vancomycin; CD: clindamycin; Mino: Minocycline; R: resistant; I: intermediate; S: susceptible

Case no.	PIT	CAZ	CPS	CPM	AT	IPM	MER	AK	GEN	CIP	LE	Col	COT	VA	CD	Mino
1	R	R	I	R	R	R	R	R	R	I	S	R	R	S	S	S
2	I	R	S	R	R	R	R	R	S	S	S	R	S	S	S	S
3	R	R	I	R	R	R	R	R	R	S	S	R	S	S	R	S
4	R	R	I	R	R	R	R	R	R	R	R	R	S	R	S	S
5	R	R	I	R	R	R	R	R	R	R	R	R	R	R	R	R
6	R	R	S	I	R	R	R	R	S	I	S	R	S	S	S	S
7	R	R	I	R	R	R	R	R	R	R	S	R	S	R	S	R
8	R	R	R	R	R	R	R	R	R	R	S	S	R	R	R	S
9	R	R	I	R	R	R	R	R	R	S	R	S	R	S	S	S
10	R	R	R	R	R	R	R	R	R	S	R	R	S	S	S	R
11	R	R	R	R	R	R	R	R	R	R	R	S	R	S	R	R
12	R	R	I	R	R	R	R	R	R	S	R	R	R	S	R	S
13	R	R	R	R	R	R	R	R	S	S	S	S	R	R	R	S
14	R	R	I	R	R	R	R	R	S	S	R	R	S	R	R	R
15	R	R	I	R	R	R	R	R	R	S	S	S	S	S	S	S
Total R (n, %)	15 (100%)	15 (100%)	0 (0%)	15 (100%)	15 (100%)	15 (100%)	15 (100%)	15 (100%)	11 (73.3%)	7 (46.7%)	7 (46.7%)	10 (66.7%)	7 (46.7%)	6 (40%)	6 (40%)	5 (33.3%)

Environmental surveillance

Following a suspected cluster of *E. meningoseptica* infections in the ICU and NICU, targeted environmental surveillance was conducted between January and March 2023, in conjunction with routine sampling, shortly before the last three clinical isolates were identified. Following HICC protocols, a total of 98 environmental samples were collected from high-risk areas, including sinks, ventilators, humidifiers, suction apparatus, ventilator trolleys, bed surfaces, cardiac monitors, tables, nebulisers, weighing machines, and other equipment. *E. meningoseptica* was isolated from one sample (1.02%), specifically from the surface of a NICU sink. Although limited, this finding suggests a possible environmental reservoir and underscores the need for stringent disinfection practices and ongoing environmental monitoring in critical care settings.

## Discussion

*E. meningoseptica* is an emerging nosocomial pathogen exhibiting intrinsic resistance to multiple antibiotics, making its treatment challenging. In our study, infections were predominantly observed in adults (80%), with a significant male preponderance (73.3%), similar to findings in previous studies [7]. The majority of infections were associated with sepsis (60%), ventilator-associated pneumonia (40%), and septic shock (40%), and consistent with reports indicating *Elizabethkingia *species as a major cause of bloodstream infections and lower respiratory tract infections [9,11].

The presence of risk factors such as hypertension (40%) and diabetes mellitus (20%) aligns with earlier reports suggesting a predisposition among individuals with underlying comorbidities [12]. Additionally, low birth weight and preterm birth were linked to both the neonatal cases in our study (100%), corroborating evidence that neonatal incubators and contaminated hospital environments act as reservoirs for this pathogen [7].

The predominant source of isolates was endotracheal (ET) secretions (46.7%), followed by tracheostomy (TT) secretions (33.33%) and blood samples (13.33%). These findings support earlier studies that identified the respiratory tract as a common site of colonization and infection [11,12]. The overall mortality rate in our study was 40%, comparable to previous reports indicating mortality rates ranging from 21% to 52% [6,7,13]. Bloodstream infections accounted for the highest mortality in adults (96%), while meningitis was the primary cause of death in pediatric cases (89%), reinforcing the clinical severity of Elizabethkingia infections [11].

Our antimicrobial susceptibility profile highlighted the multidrug-resistant nature of *E. meningoseptica*, with 100% resistance to carbapenems (imipenem, meropenem), cephalosporins (ceftazidime, cefepime), aztreonam, and piperacillin-tazobactam. This resistance is attributed to the production of metallo-beta-lactamases (BlaB and GOB genes) [14]. High resistance was also noted for gentamicin (73.33%), colistin (66.67%), and cotrimoxazole (46.7%), further limiting therapeutic options. However, minocycline (66.7%), vancomycin (60%), and levofloxacin/Ciprofloxacin (53.3%) showed relatively better susceptibility, making them potential treatment choices. Similar findings have been reported in other studies, where minocycline and piperacillin-tazobactam demonstrated high efficacy against *Elizabethkingia* species [15,16].

In our study, *E. meningoseptica* was isolated from the handwash sink in the NICU, suggesting it may act as an environmental reservoir, consistent with previous reports that highlight the organism’s ability to colonize moist hospital surfaces such as sink basins and taps [17]. Its intrinsic resistance to chlorination allows it to persist in treated water supplies, posing a risk for nosocomial transmission. This raises concerns about possible transmission via contaminated water sources and lapses in infection control practices, particularly regarding hand hygiene and aseptic techniques.

Colonization of patients may occur through contact with contaminated surfaces or indirectly via healthcare workers, with subsequent progression to infection. This underlines the importance of heightened clinical suspicion and timely microbiological identification. Once *E. meningoseptica* is suspected, it is imperative for microbiologists to alert clinicians promptly, enabling early initiation of appropriate antimicrobial therapy.

Given the organism’s multidrug resistance and environmental resilience, clinicians must remain vigilant and consider it as a potential pathogen, especially in critically ill or device-associated patients. Effective infection control interventions as routine water quality surveillance, regular disinfection of hospital sinks and water outlets, and hyperchlorination of water systems-may be necessary to prevent further outbreaks. Further research is warranted to better understand the epidemiology, risk factors, and resistance mechanisms associated with *E. meningoseptica *and to develop targeted strategies for its management and control.

This study has several limitations. First, the retrospective design restricts control over confounding variables and limits the ability to establish causal relationships. Second, the small sample size (n = 15) reduces the statistical power, precludes inferential analysis, and limits the generalizability of findings. Third, reliance on retrospective medical records may have led to incomplete capture of key clinical variables, including the timing of *E. meningoseptica* isolation relative to admission, prior antimicrobial exposure, changes in therapy post-culture, and repeat culture data, hindering a comprehensive understanding of treatment impact and organism persistence. Additionally, the exclusion of polymicrobial cases may introduce selection bias. Finally, environmental surveillance was limited to targeted high-risk ICU/NICU sites over a defined period during a suspected outbreak, which may not fully reflect broader environmental contamination patterns. These limitations underscore the need for larger, prospective studies with standardized microbiological protocols, systematic follow-up, and analytical frameworks to better define the clinical relevance and transmission dynamics of this emerging pathogen.

## Conclusions

*E. meningoseptica* is an emerging multidrug-resistant pathogen associated with severe infections, particularly in critically ill patients with risk factors such as prolonged hospitalization and mechanical ventilation. In our study, sepsis was the most common presentation (60%), and the overall mortality rate was 40%, underscoring its clinical significance. All isolates demonstrated resistance to carbapenems, beta-lactams, and aminoglycosides, with the highest susceptibility to minocycline (66.7%), vancomycin (60.0%), and levofloxacin (53.3%). These findings highlight the importance of early species-level identification using automated systems like Vitek-2 to guide appropriate therapy.

As part of targeted surveillance prompted by a suspected ICU/NICU outbreak, *E. meningoseptica* was isolated from one of 98 environmental samples (1.02%), indicating a potential environmental reservoir and reinforcing the need for rigorous infection prevention strategies. Given the small sample size, inferential statistical analysis was not feasible. Future prospective studies with larger cohorts are warranted to better define risk factors, treatment outcomes, and resistance mechanisms. Continued environmental monitoring and antimicrobial stewardship remain critical to limiting nosocomial spread and improving patient outcomes.
